# The Railway “Boss” Surgeons and the Medical Profession

**Published:** 1889-06

**Authors:** 


### THE RAILWAY "BOSS" SURGEONS AND THE MEDICAL PROFESSION.

At the recent Convention of Railway Surgeons, held in St. Louis, there was a very large attendance, some five hundred physicians more or less connected with railway interests being present.

The "policy," if it may be designated by that name, which common report says has obtained to a great extent, of offering a reputable physician a "pass" as compensation for any and all professional service he may render employees of a road under orders of the chiefs—if he accept the appointment of local surgeon of a road—received a decided "black eye," if we may judge by reports of the meeting in the Kansas Medical Journal. That such offers have been made, there is no lack of proof. We have been informed by a prominent physician in Austin that certain railroad surgeons have boasted that "there is no trouble in getting a doctor to accept the position of local surgeon, for the *eclat* it gives him, and that he is generally satisfied if a pass be thrown in; that if one will not accept such offer, another will." That is to say, there is no trouble in finding a practicing physician who is willing to go, day or night, to anybody connected with the railroad, on orders of the chief, and render such services as may be required, *free of charge;* or, if he make a charge, it is tacitly understood that it is not to be paid in money—he has a pass! That such offers are not always accepted, be it said to the credit of the profession, one gentleman at the meeting testified. He said that such "contract" had been offered him, and it had been "declined with thanks," and "what do you take me for?"

To such as cannot be gulled by a pass and an allowance of *eclat*, a contract is offered with the price fixed for services in advance—so much a visit by day, and so much for night visits; so much for fractures, so much for operations of any and all kinds, and that price is generally less than half usually charged by said doctor in his private practice.

That there are men in the profession who are willing to work for rich corporations for less than half price, is a lamentable fact; that there are many who have the manhood and dignity to refuse such offers, is also evident.

It was an acquaintance with the prevalence of this compromising custom which induced delegate Swearingen from the Austin District Medical Society to introduce at the late meeting of the Texas State Medical Association, at San Antonio, and the Association to adopt, unanimously, by a rising vote, the follow-resolution:

"A chedule of charges that requires a physician to charge a railway corporation from fifty to one hundred per cent. less than he would charge his neighbor for the same kind of service, insults our sense of justice and violates the spirit of our ethics."

It was the intention to place on the desk of each delegate at St. Louis a copy of the resolutions from which the above is extracted, (see DANIEL'S TEXAS MEDICAL JOURNAL for May, '89,) but owing to a misunderstanding as to the date of meeting, it was not done. However, it has been pretty generally distributed, and a copy has been sent especially to railroad "boss" surgeons.

It is earnestly hoped, for the honor and dignity of the profession, that we will hear no more of a reputable physician committing himself to a contract to work for a railroad company at their own price, which price is, as stated, less than half usually charged others; and especially that we will hear no more of selling their services for a "mess of pottage." Indeed, it savors much of a species of assurance commonly denominated in the vernacular as "cheek," to approach a physician with such an offer as a pass, in compensation for services *to be* performed; and what are we to say of the physician who accepts such an offer? Surely, he puts a low valuation on his services, and yet it is not always a case of "poor preach, poor pay," for some excellent surgeons are thus employed, we understand.

To the gentleman who, at St. Louis, made public the fact that such practice obtains, and that he had been thus approached, and to Dr. Swearingen for introducing the resolution referred to, the thanks of the profession are due.

Dr. Cross' name was dropped from the roll of the Alabama Medical Association for "unprofessional conduct," because he entered into a contract to practice for a corporation. He is suing the Association for $50,000 damages. So this is a serious matter.

---

THE MEDICO-LEGAL SOCIETY, of Chicago, elected Dr. E. J. Doering president, at last meeting.

